# DNA repair proteins may differentiate papillary thyroid cancer from chronic lymphocytic thyroiditis and nodular colloidal goiter

**DOI:** 10.1038/s41598-021-89403-0

**Published:** 2021-05-11

**Authors:** Bahri Evren, Sami Yılmaz, Neşe Karadağ, Ayşe Çıkım Sertkaya, Ömercan Topaloğlu, Faruk Kılınç

**Affiliations:** 1 Department of Endocrinology and Metabolism, Inonu University School of Medicine, Malatya, Turkey; 2 Department of İnternal Medicine, Yayladağ State Hospital, Hatay, Turkey; 3 Department of Pathology, Inonu University School of Medicine, Malatya, Turkey; 4 Department of Endocrinology and Metabolism, American Hospital, İstanbul, Turkey; 5 Medical School, Department of Endocrinology and Metabolism, Zonguldak Bülent Ecevit University, Zonguldak, Turkey; 6 Department of Endocrinology and Metabolism, Fırat University Medical School, Elazığ, Turkey

**Keywords:** Endocrinology, Pathogenesis

## Abstract

Malignant thyroid lesions are the most common malignancy of the endocrine glands with increasing rates in the last two decades. Papillary thyroid cancer is the most common thyroid malignancy. In our study, we aimed to quantitatively evaluate the levels of DNA repair proteins MSH2, MLH1, MGMT, which are representative blocks of patients diagnosed with papillary carcinoma, chronic thyroiditis, or colloidal goiter. Total or subtotal thyroidectomy material of 90 patients diagnosed with papillary carcinoma, nodular colloidal goiter, or chronic thyroiditis between 2009 and 2012 were retrospectively evaluated. Tissue samples obtained from paraffin blocks were stained with MGMT, MSH2, MLH1 proteins and their immunohistochemistry was evaluated. Prepared sections were examined qualitatively by an impartial pathologist and a clinician, taking into account the staining method under the trinocular light microscope. Although there was no statistically significant difference in MGMT, MSH2, MLH1, follicular cell positivity, staining intensity, and immunoreactivity values, papillary carcinoma cases showed a higher rate of follicular cell positivity, and this difference was more pronounced between papillary carcinoma and colloidal goiter. In the MSH2 follicular cell positivity evaluation, the difference between chronic thyroiditis and colloidal goiter was significant (p = 0.023). The difference between chronic thyroiditis and colloidal goiter was significant in the MSH2 staining intensity evaluation (p = 0.001). The difference between chronic thyroiditis and colloidal goiter was significant in MLH1 immunoreactivity evaluation (p = 0.012). Papillary carcinoma cases were demonstrated by nuclear staining only for MSH2 and MLH1 proteins as opposed to hyperplastic nodules. The higher levels of expression of DNA repair genes in malignant tumors compared to benign tumors are attributed to the functional activation of DNA repair genes. Further studies are needed for DNA repair proteins to be a potential test in the development and progression of thyroid cancer.

## Introduction

Chronic lymphocytic thyroiditis (CLT) is a common autoimmune thyroid disease in which the thyroid gland is attacked by various antibodies and cell-mediated immune processes. Dailey et al. in 1955, reported the coexistence of CLT in thyroid cancer for the first time. The role of CLT as a prognostic factor in thyroid cancer is controversial, but it is known that PTC is approximately three-fold in the presence of CLT. Recent studies have reported an association between Hashimoto's thyroiditis, BRAFV600E mutation, and clinicopathological features in patients with PTC. However, this issue is still controversial, as more studies are needed on the correlation between the BRAFV600E mutation as a prognostic factor for CLT and PTC^1^. Colloid (or nodular) goiter is an increase in the volume of the thyroid gland caused by hyperplasia of the parenchyma and leads to a state of excessive follicular proliferation. The classification of goiter is defined as diffuse and nodular and is single or multinodular. According to thyroid hormone production, anatomic-clinical separation is done as toxic (or hyperactive) and non-toxic. The classification can also be endemic or sporadic, given endemic goiter affecting more than 10% of the population in a given geographic region and sporadic goiter caused by several environmental, immunological, and genetic factors that interfere with hormone synthesis^2^.

The prediction of malignancy of thyroid nodules continues to improve. Sonographic features of thyroid nodules alone are not sufficient to predict malignancy risk^3^. Each year, as many as 50,000 patients in the United States undergo unnecessary thyroidectomy because of the difficulty in distinguishing benign thyroid nodules from thyroid cancers preoperatively. It is estimated that up to 70% of thyroidectomies are unnecessary. Papillary thyroid cancer (PTC) represents about 75–80% of all thyroid cancers. The diagnosis of thyroid cancer is made by fine-needle aspiration (FNAB) biopsy from thyroid nodules. Unlike many cancers, diagnosis of well-differentiated thyroid cancer is difficult due to the frequent overlap of cytological features between malignant and benign nodules from FNAB. Molecular tests have begun to be used for thyroid nodules with uncertain cytology observed at rates of up to 40%. DNA methylation is a stable epigenetic modification that plays an important role in cancer initiation and progression. Studies comparing thyroid cancer with neighboring thyroid tissues revealed that DNA methylation patterns in PTC are strongly associated with mRNA and miRNA signatures, BRAFV600E and H/K/NRAS mutations, and often reflect a histological tumor type^4^. Microsatellite instability (MSI) is the accumulation of repetitive insertion or deletion mutations in DNA sequences mainly due to defects in DNA mismatch repair (MMR). The MSI status may be due to the detection of the expression of the MMR proteins MLH1, PMS2, MSH2, and MSH6 via immunohistochemistry (IHC) because MSI is caused by fundamental defects in the DNA MMR. While MSI is widely reported in colorectal, endometrial, and stomach cancers, the prevalence of MSI in thyroid cancer appears to be low, but a large and detailed study is lacking. Previous research on MSI in thyroid cancer was almost entirely done in PTC cases, and less common histological subtypes were neglected^4^. To date, few case–control studies have been conducted on radiation-related PTC, and published data show that changes in expression levels and polymorphisms in some DNA repair genes belonging to different pathways are associated with the risk of developing thyroid cancer^5^. Changes in the expression levels of DNA repair genes have been associated with an increased risk of developing thyroid cancer. Among them, MMR proteins such as MSH2 (Mut-S-Homologin-2) and MLH1 (Mut-L-Homologin-1) have been implicated in the development and progression of various head and neck neoplasms, including the thyroid. Loss of MGMT expression has been associated with hepatocellular, lung, stomach, and breast carcinomas, aggressive tumor behavior, and progression in various types of neoplasia, including the esophagus^6^. However, available data evaluating the immunohistochemical expression of MSH2, MLH1, and MGMT in benign and malignant thyroid lesions have so far been insufficient.

In this study, we aim to retrospectively measure the levels of DNA repair proteins (MSH2, MLH1, and MGMT) from representative blocks of tissue material in patients with a confirmed diagnosis of papillary thyroid carcinoma, chronic thyroiditis, or nodular colloidal goiter by thyroidectomy and to examine the clinical predictive effect of these levels.

## Materials and methods

Total or subtotal thyroidectomy material belonging to 90 adult patients who were diagnosed as papillary carcinoma, nodular colloidal goiter, and thyroiditis between 2009 and 2012, which were in the archive of İnönü University Medical Faculty Pathology Department Laboratory, were evaluated retrospectively. Representative tissue blocks were selected by re-evaluating all sections stained with Hematoxylin–Eosin of the cases. Cases with poorly differentiated, undifferentiated carcinomas, follicular and medullary carcinomas were excluded. Selected blocks were stained with MGMT, MSH2, MLH1 proteins, and immunohistochemistry was evaluated. 6 case preparations were excluded due to technical problems in staining. The patients were divided into 3 groups: papillary thyroid carcinoma (PTC, n = 29), chronic thyroiditis (n = 29) or nodular colloidal goiter (n = 26). We evaluated papillary thyrooid cancer as regards to cancer staging, focality and invasion properties of the cancer^7^. Ethics committee approval was obtained from the ethics committee of our university with the number 2012/38.

### Immunohistochemical staining

After fixing the tissues to be evaluated in 10% neutral buffered formalin, they were embedded in paraffin following performing routine tissue follow-up. The sections taken at 3–5 μm thickness were kept in the oven at 56 °C for 15 min. Preparations 1—Mismatch Repair Protein (MSH2) MSH2-CE, CLONE: 25D12, 2—Mismatch Repair Protein (MLH1) MLH1-L-CE, CLONE: ES05, and 3—MGMT ab88764, were stained with immune stains by using an automated immunohistochemistry device, Bond-maX (Leica Microsystems). By using Bond Polymer Refine Detection Kits (Leica, DS9800), the following operations were performed respectively:

1. Deparaffinization (Bond Dewax Solution, Leica Microsystems).
2. Rehydration (alcohol).
3. Wash (Bond Wash Solution, Leica Microsystems).
4. Removal of antigenic masking (Bond Epitop Retrieval Solution, Leica Microsystems).
5. Washing (Bond Wash Solution, Leica Microsystems).
6. Inhibition of endogenous peroxidase activity (Peroxide Block, Leica Microsystems).
7. Wash (Bond Wash Solution, Leica Microsystems).
8. Primary antibody (anti-eNOS; Genetex Inc., Irvine, California, anti-iNOS; 1: 100 dilution, Genetex Inc., Irvine, California).
9. Washing (Bond Wash Solution, Leica Microsystems).
10. Secondary antibody (Post Primary, Leica Microsystems).
11. Washing (Bond Wash Solution, Leica Microsystems).
12. Polymer (Leica Microsystems).
13. Washing (Bond Wash Solution, Leica Microsystems).
14. Washing (distilled water).
15. Color reaction with DAB (3,3′-diaminobenzidine tetrahydrochloride) (Mixed DAB Refine, Leica Microsystems).
16. Washing (distilled water).
17. Contrast staining (Mayer hematoxylin).
18. Washing (distilled water).
19. Washing (Bond Wash Solution, Leica Microsystems).
20. Washing (distilled water) operations were carried out automatically in the device.

The stained preparations were covered with a lamella using water-based covering gel.

### Immunohistochemical evaluation

Prepared sections were qualitatively examined by an impartial pathologist, taking into account the staining method under the trinocular light microscope (Olympus BX-51). The evaluation method in the preparation is based on a previous study and given below^6^.

#### Follicular cell positivity

- 0: 0–4% follicular cell positivity (negative staining).
- 1: 5–24% follicular cell positivity.
- 2: 25–49% follicular cell positivity.
- 3: 50–100% follicular cell positivity.

#### Staining intensity

- 0: negative staining.
- 1: light staining.
- 2: medium staining.
- 3: intense staining.

#### Immunoreactivity assessment

According to follicular cell positivity and staining intensity scores;

- 0–2: negative/weak.
-  ≥ 3: medium/strong.
- Staining pattern.
- Nuclear.
- Cytoplasmic.
- Nuclear-cytoplasmic.

**Figure 1:**
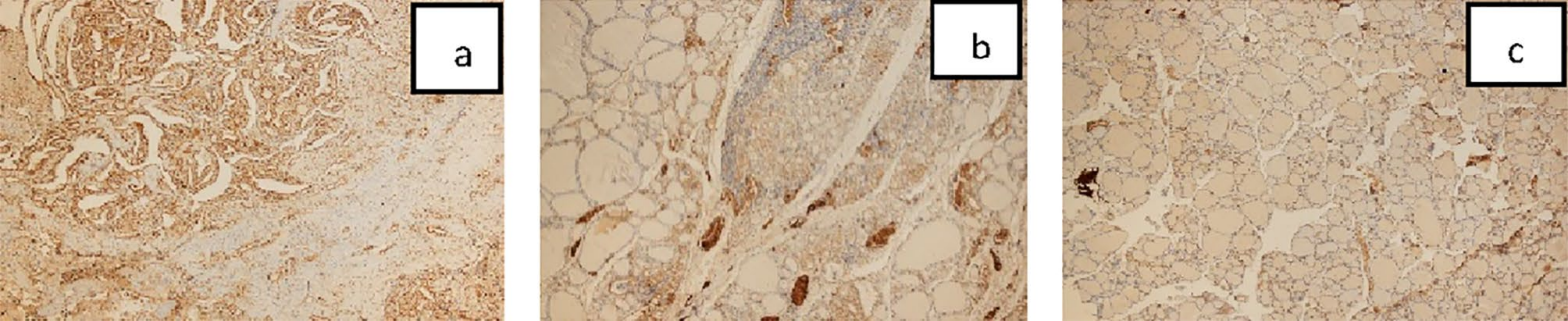
(**a**) MGMT immunoreactivity PTC (× 4). (**b**) MGMT immunoreactivity Chronic thyroiditis (× 4). (**c**) MGMT immunoreactivity Nodular colloidal goiter (× 4).

**Figure 2:**
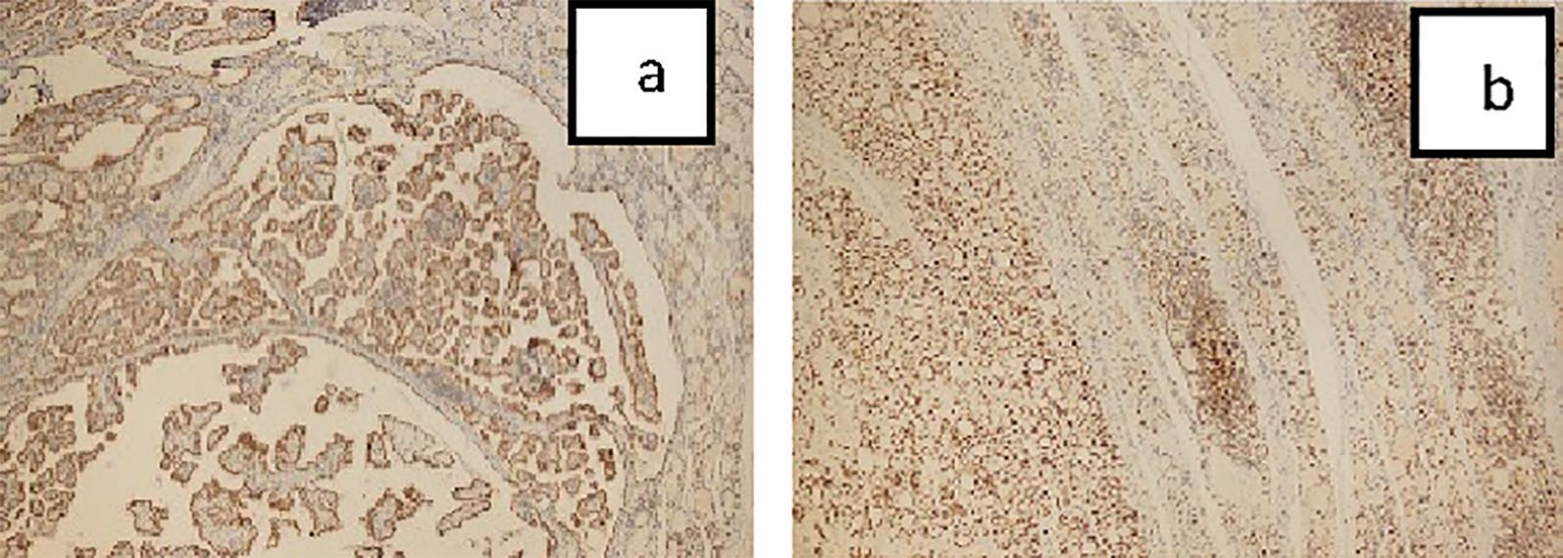
(**a**) MSH2 immunoreactivity PTC (× 4). (**b**) MSH2 immunoreactivity Chronic thyroiditis (× 4).

**Figure 3:**
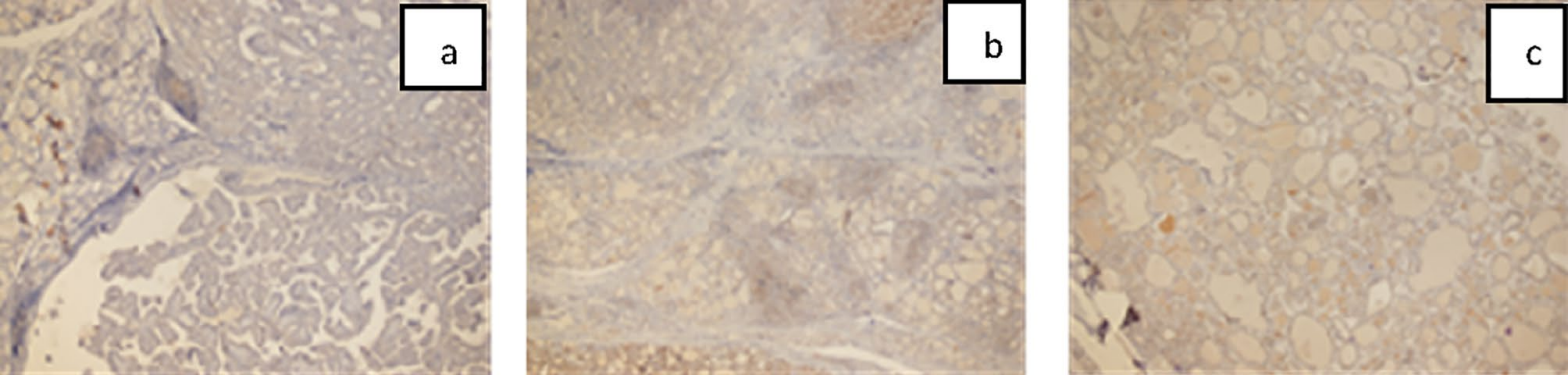
(**a**) MLH1 immunoreactivity PTC (× 4). (**b**) MLH1 immunoreactivity Chronic thyroiditis (× 4). (**c**) MLH1 immunoreactivity Nodular colloidal goiter (× 4).

MGMT, MSH2, MLH1 immunoreactivity values are given in Figs. 1, 2 and 3 under the microscope at × 4 magnification.

### Statistical analysis

Statistical evaluation was performed with the SPSS (Statistical Package for Social Scienses) 16.0 program. Descriptive statistical methods (mean, standard deviation) were used while evaluating the study data, and ANOVA test was used to evaluate quantitative data. Pearson Chi-Square test was used to evaluate qualitative data. Chi-square test was used to evaluate categorical parameters between groups; since the comparison cannot be made when the two cell contents are < 5, only the ratios are given for the groups with this feature. Fisher's Exact Test was applied for intergroup evaluation in groups with suitable conditions. The results were evaluated at the 95% confidence interval and the significance level at p < 0.05.

### Ethics approval

We performed our study in accordance with the ethical standards laid down in the 1964 Declaration of Helsinki and its later amendments. Our study was approved by Local Ethics Committee (Malatya Clinical Research Ethics Committee) with an approval number of 2012/38.

### Consent to participate

All subjects did give informed consent to participate in research.

## Results

The patients were divided into 3 groups: papillary thyroid carcinoma (PTC, n = 29), chronic thyroiditis (n = 29) or nodular colloidal goiter (n = 26). The mean ages of the groups were similar (p = 0.26). The rate of autoimmune markers was significantly higher in the chronic thyroiditis group (p < 0.001) (Table 1). The number of the patients with PTC was 12 in stage 1, 11 in stage 2, and 6 in stage 3. A total of 11 patients with PTC did have multifocal cancer, and lymphovascular invasion was detected in 7 patients.

**Table 1:** Demographic characteristics of patient groups and comparison of autoimmunity.

	PTC (n = 29)	Chronic thyroiditis (n = 29)	Nodular collodidal goiter (n = 26)	p
Age (mean ± SD.)	50.07 ± 16.42	46.21 ± 11.80	52.96 ± 17.09	0.260
Female/male (%)	69/31	100/0	81/19	
AntiTPO (%)	5.3	50	6.2	< 0.001
AntiTG (%)	5.3	46.2	7.7	< 0.001

When MGMT, MSH2, MLH1 were evaluated in terms of 50–100% follicular cell positivity, although there was no statistically significant difference, papillary carcinoma cases showed a higher rate of follicular cell positivity, as seen in Table 2, and this difference was more pronounced between papillary carcinoma and colloidal goiter.

**Table 2:** A 50–100% follicular cell positivity rates of MGMT, MSH2, MLH1 groups.

Follicular cell positivity (50–100%)	PTC (n = 29) (%)	Chronic thyroiditis (n = 29) (%)	Nodular colloidal goiter (n = 26) (%)	p
MGMT	86.2	82.8	76	> 0.005
MSH2	86.2	72.4	42.3	> 0.005
MLH1	55.2	51.7	26.9	> 0.005

Although there was no statistically significant difference in MGMT and MSH2 staining intensity, as seen in Table 3, there was no difference between papillary carcinoma cases and chronic thyroiditis cases in terms of intense staining, but the difference between papillary carcinoma and colloidal goiter was more pronounced in favor of papillary carcinoma. In the evaluation of MSH2 staining intensity, as seen in Table 3, if a double examination was made in the form of light staining and medium-intense staining, it was found that the difference between chronic thyroiditis and colloidal goiter was statistically significant (p = 0.001).

**Table 3:** Staining intensity ratios of MGMT, MSH2, MLH1 groups.

Staining intensity	PTC (n = 29)	Chronic thyroditis (n = 29)	Nodular colloidal goiter (n = 26)
**MGMT**			
Mild (%)	13.8	6.2	8
Medium (%)	48.3	55.2	64
Intensive (%)	37.9	37.9	28
**MSH2**			
Mild (%)	20.7	13.8	80.8
Medium (%)	75.9	82.8	19.2
Intensive (%)	3.4	3.4	0
**MLH1**			
Mild (%)	6.9	0	15.4
Medium (%)	82.8	75.9	69.2
Intensive (%)	10.3	24.1	15.4

Although there was no statistically significant difference in terms of MGMT, MSH2, MLH1 immunoreactivity values, as seen in Table 4, there was no difference in immunoreactivity between papillary carcinoma cases and chronic thyroiditis cases, but the difference between papillary carcinoma and colloidal goiter was more pronounced in favor of papillary carcinoma. The difference between chronic thyroiditis and colloidal goiter was found to be significant in MSH2 immunoreactivity evaluation (p = 0.044). In MLH1 immunoreactivity evaluation, the difference between chronic thyroiditis and colloidal goiter was found to be significant (p = 0.012).

**Table 4:** Immunreactivity ratios of MGMT, MSH2, MLH1 groups.

Immunoreactivity rates	PTC (n = 29)	Chronic thyroditis (n = 29)	Nodular colloidal goiter (n = 26)
**MGMT**			
Negative/weak (%)	0	0	8
Medium/strong (%)	100	100	92
**MSH2**			
Negative/weak (%)	3.4	0	15.4
Medium/strong (%)	96.6	100	84.6
**MLH1**			
Negative/weak (%)	6.9	0	15.4
Medium/strong (%)	93.1	100	84.6

Although there was no statistically significant difference in terms of MGMT staining types, as seen in Table 5, although there was no significant difference in MGMT staining patterns between papillary carcinoma cases and chronic thyroiditis cases, papillary carcinoma cases mostly showed nuclear and cytoplasmic, colloidal goiter cases mostly showed nuclear staining. All groups showed nuclear staining with MSH2. The difference between MLH1 staining type chronic thyroiditis and colloidal goiter was found to be significant (p = 0.044). Although there was no statistically significant difference in the type of staining with MLH1, the rate of negativity was higher in colloidal goiter cases.

**Table 5:** Staining types of MGMT, MSH2, MLH1 groups.

Staining types	PTC (n = 29)	Chronic thyroditis (n = 29)	Nodular colloidal goiter (n = 26)
**MGMT**			
Nuclear (%)	65.5	62.1	84
Nuclear + cytoplasmic (%)	34.5	37.9	16
**MSH2**			
Nuclear (%)	100	100	100
Nuclear + cytoplasmic (%)	0	0	0
**MLH1**			
No staining (%)	6.9	0	15.4
Nuclear (%)	93.1	100	84.6

## Discussion

Most thyroid cancers are papillary carcinomas in adults and children. Diagnostic, therapeutic, and environmental exposure are well-known risk factors for thyroid cancers^8^. Radiation exposure in childhood is an important risk factor in PTC etiology. Polymorphisms in DNA repair genes are likely to affect this risk, but few studies have been designed to determine the role of such genes as modulators of PTC risk^5^. It is known that inherited or acquired deficiencies in DNA repair proteins can lead to deleterious mutations, cell death associated with cancer development, differentiation, and progression. In particular, changes in the expression levels and polymorphisms of DNA repair genes have been considered responsible for thyroid carcinogenesis^6^. There are limited studies in the literature on the importance of IHC expressions of MSH2, MLH1, and MGMT in the differentiation of benign and malignant thyroid lesions.

In the study of Giaginis et al., MGMT expression levels were found to be lower in malignant thyroid lesions compared to benign cases. Papillary carcinoma cases also show an increased incidence of low MGMT compared to hyperplastic nodules. Moreover, low MGMT is associated with large tumor size in thyroid cancer cases^6^. In addition, Schagdarsureng reported that MGMT hypermethylation is occurred priorly compared to differentials in undifferentiated thyroid carcinomas^9^. Loss of MGMT expression in some cancer tissues such as hepatocellular, stomach, esophagus, and bile ducts is correlated with clinicopathological parameters and poor prognosis^6^. Loss of MGMT expression in patients with oral squamous cell carcinoma is associated with advanced stage, lymph node positivity, and poor prognosis. Significant loss of MGMT expression in precancerous oral lesions has been demonstrated from hyperplasia to dysplasia, supporting the hypothesis of MGMT disorder in the case of early oral tumorigenesis^10^. Low MGMT expression is correlated with poor prognosis and hepatic invasion in carcinoma of the biliary tract. Hepatocellular carcinoma patients with decreased MGMT were observed to have a poor prognosis and advanced disease stage. Again, low MGMT expression in gastric cancer patients is associated with serosal invasion, advanced-stage disease, lymph node positivity, undifferentiated histopathological type, and poor prognosis. Decreased MGMT protein expression levels in malignant thyroid lesions may result in tumor progression and/or progression of thyroid neoplasia, in line with evidence from other types of cancer. Within the MMR proteins, MLH1 expression levels were shown to increase in the frequency of moderate/strong MLH1 expression in papillary carcinoma cases compared with benign thyroid lesions, especially hyperplastic nodules. MSH2 expression was not significant in differentiating malignant and benign thyroid lesions^6^. Ruschenburg et al. reported that the expression levels of 3 MMR proteins MLH1, MSH2, and PMS1 generally increase in malignant thyroid lesions compared to benign ones^11^. The difference in expression levels of MSH2 and MLH1 may result in tumor growth and/or progression in thyroid cancers. In this respect, the connection between methylation of the MLH1 gene and lymph node metastasis in patients with papillary thyroid carcinoma with T1799A BRAF mutation has been reported as epigenetic differentiation of MLH1^12^. In our study, in the evaluation of MGMT, MSH2, and MLH1 antibody staining, follicular cell positivity, and staining density were also evaluated separately and the results were determined. When MGMT, MSH2, MLH1 were evaluated in terms of 50–100% follicular cell positivity, although there was no statistically significant difference, papillary carcinoma cases showed a higher rate of follicular cell positivity, and this difference was more pronounced between papillary carcinoma and colloidal goiter. The difference between chronic thyroiditis and colloidal goiter was significant in the dual statistical evaluation performed as less than 50% follicular cell positivity and 50% or more follicular cell positivity in MSH2 follicular cell positivity (p = 0.023).

In papillary carcinoma cases, the frequency of moderate/high MSH2 immunoreactivity was increased compared to hyperplastic nodules, but it was not statistically significant. It has been documented that moderate/strong MSH2 and MLH1 immunoreactivity is more common in cases with improved follicular cell proliferation capacity. This finding shows that MMR proteins can be interpreted as an increase in the necessity of the DNA repair system in rapidly proliferating follicular cells in the case of cell proliferation in thyroid neoplasms^6^. In our study, although there was no difference between papillary carcinoma cases and chronic thyroiditis cases in terms of MGMT, MSH2, and immunoreactivity values, the difference between papillary carcinoma and colloidal goiter was more pronounced in favor of papillary carcinoma. The difference between chronic thyroiditis and colloidal goiter was found to be significant in MSH2 immunoreactivity evaluation (p = 0.044). In MLH1 immunoreactivity evaluation, the difference between chronic thyroiditis and colloidal goiter was found to be significant (p = 0.012). Although there was no statistically significant difference between the other groups, the MLH1 immunoreactivity value between papillary carcinoma cases and chronic thyroiditis cases was significant in favor of chronic thyroiditis, while the difference between papillary carcinoma and colloidal goiter was more pronounced in favor of papillary carcinoma.

Staining of malignant thyroid lesions with nuclear distribution in the nucleus and follicular cell cytoplasm with MGMT, unlike hyperplastic nodules, can be attributed to MGMT gene mutation as in other types of cancer^13^. A similar distribution pattern can be observed in cases with Hashimoto's thyroiditis. These cases almost always involve genetic rearrangement. These cases are strongly linked to papillary thyroid carcinoma and increase the likelihood of a molecular link between the two diseases^14^. Different cellular localization of MMR proteins was recorded. Nuclear and cytoplasmic distribution of papillary carcinoma cases for MSH2 and MLH1 proteins was demonstrated by nuclear staining only, in contrast to hyperplastic nodules. These findings may show that; While MMR proteins cannot bind to nuclear DNA anymore, destabilization of the MMR protein complex can be progressive from hyperplasia to malignancy. Both nuclear and cytoplasmic MSH2 and MLH1 protein distribution have been shown in cases of Hashimoto's thyroiditis. This shows that the morphological features, immunohistochemical patterns, and most importantly, the molecular profile of papillary thyroid cancer and Hashimoto thyroiditis coincide. Although considered to be benign, almost all Hashimoto thyroiditis are genetically remodeled, always harboring papillary thyroid carcinoma with a strong and high specificity^6^. In our study, although there was no significant difference in terms of MGMT staining patterns, papillary carcinoma cases mostly showed nuclear and cytoplasmic staining colloidal goiter cases showed mostly nuclear staining, similar to the literature^6^. In our study, the MSH2 staining pattern was nuclear staining in all groups. MLH1 staining type was 100% nuclear in chronic thyroiditis cases, and 85% nuclear, 15% nuclear, and cytoplasmic in cases of colloidal goiter, and the difference was found to be significant (p = 0.044). Although there was no statistically significant difference between the other groups in terms of staining pattern, the rate of negativity was higher in colloidal goiter cases.

In a meta-analysis by Xingjian Lai et al., Heterogeneity was found among studies, but it was shown that PTC was more common in patients with Hashimoto's thyroiditis than patients without Hashimoto's thyroiditis. In the study of Anıl et al., The prevalence of thyroid carcinoma was compared prospectively among 164 patients with Hashimoto's thyroiditis and 551 patients without Hashimoto's thyroiditis and found that thyroid nodules in patients with Hashimoto's thyroiditis were not different from those without Hashimoto's thyroiditis^15^.

The evaluations made in our study also showed that; DNA repair genes did not differ significantly in patients with papillary thyroid carcinoma and chronic thyroiditis.

The weaknesses of our study may be the small number of patients evaluated, not evaluating the nodule sizes of the evaluated patients, and we did not include subtypes of PTC cases in the study. Differences could be found between PTC subtypes in terms of expression of DNA repair proteins. By analyzing the clinical findings and preoperative laboratory parameters of PTC cases, their relationship with DNA repair proteins could be examined. Similarly, analyzing the cases in the three groups, other clinical findings, and preoperative serum autoimmune markers could be helpful in understanding the differences or similarities between the groups. There are limited studies in the literature that include cases of PTC, chronic thyroiditis, and nodular colloidal goiter, and analyzes DNA repair proteins with IHC. Our findings are valuable in this respect.

As a result, the expression of DNA repair genes in malignant tumors at higher levels compared to benign tumors is attributed to functional activation in DNA repair genes due to DNA damage that occurs as a result of malignant transformation. Our study shows us that changes in the expression levels of MMR and MGMT proteins may result in tumor growth and/or progression of thyroid neoplasia. Larger cohort studies are recommended to improve the diagnostic investigation in thyroid neoplasms and to more precisely demonstrate the clinical significance of DNA repair proteins. In future studies, the utility of the molecular mechanism of DNA repair proteins as a potential test in the development and progression of thyroid cancer.

## Data Availability

The datasets generated during and/or analyzed during the current study are available from the corresponding author on reasonable request.
